# Electrochemotherapy in Kaposi’s Sarcoma Patients: From the Gold Standard Strategy to Locally Advanced Cutaneous and Subcutaneous Lesions

**DOI:** 10.3390/cancers16071295

**Published:** 2024-03-27

**Authors:** Vincenzo Rullo, Francesco Castellaneta, Santolo D’Antonio, Anna De Rosa, Michele Pio Grieco, Tommaso Fabrizio

**Affiliations:** 1 Division of Plastic Surgery, IRCCS-Centro di riferimento Oncologico della Basilicata, Via Padre Pio, 1, 95098 Rionero in Vulture, Italy; vincenzo.rullo2@unina.it (V.R.); francesco.castellaneta@crob.it (F.C.); santolo.dantonio@unina.it (S.D.); anna.derosa4@unina.it (A.D.R.); michele.grieco@crob.it (M.P.G.); 2 Division of Plastic Surgery, AOU Federico II, Via S. Pansini, 5, 80131 Naples, Italy

**Keywords:** electrochemotherapy, Kaposi’s sarcoma, electroporation, angio-proliferative malignancy, cutaneous and subcutaneous lesions

## Abstract

**Simple Summary:**

Electrochemotherapy (ECT) should be considered a valid therapeutical strategy for the local control of widespread and advanced CKS cutaneous and subcutaneous lesions. The aim of our study is not only to validate and confirm that ECT represents the best therapeutical choice in terms of the risk–benefit ratio for the treatment of cutaneous and subcutaneous lesions in non-advanced forms of Kaposi’s sarcoma, but also to demonstrate the valid use of ECT for the local control of locally advanced classic Kaposi’s sarcoma (CKS). Among 19 patients treated, acceptable results have also been obtained in those patients with widespread CKS lesions due to the silent course of the KS classic variant and the excellent impact of the disease on quality of life.

**Abstract:**

Electrochemotherapy (ECT) is one of the newest therapeutic strategies employed as a medical procedure for skin neoplasms’ treatment, especially for classic Kaposi’s sarcoma (CKS). The aim of this study was to demonstrate ECT clinical response and the local control of CKS disease. The primary endpoint was to value the worth and efficacy of this local therapy in CKS skin lesions’ treatment. In total, 19 CKS patients were enrolled, 14 males and 5 females with median age at diagnosis of 72. Complete response (CR) has been gained in 12 patients after first ECT attempt; meanwhile, 3 and 4 out of 19 patients obtained a partial response (PR), so they underwent a second and third ECT treatment, respectively. Clinical response was evaluated during the entire timeframe of the follow-up, which ranged between 3 months and 4 years with a median of 18 months. The control of CKS skin lesions still represents a challenge for surgeons and oncologists. Nevertheless, according to this and other authors’ recent experiences, ECT could be considered the gold standard strategy for early-stage patients, but at the same time it could be considered as a valid option in controlling Kaposi’s sarcoma locally advanced lesions.

## 1. Introduction

Kaposi’s sarcoma (KS) is an angio-proliferative malignancy, involving blood and lymphatic vessels, strongly related to infection with human herpesvirus 8 (HHV8), also known as Kaposi’s sarcoma-related herpesvirus (KSHV); HHV-8 infection is a necessary but not sufficient condition to cause KS. Thus, other factors such as immunosuppression have been demonstrated to play an important role in KS pathogenesis [1,2]. Although four different clinical–epidemiological variants were described, the classic form of KS is localized on the skin and subcutaneous tissues of the lower limbs (followed by upper limbs, head, and trunk) with purplish maculo-papular lesions, which can rapidly evolve into multicentric and ulcerated plaques, or nodules, often associated with venous stasis, lymphedema, and pain. Most of the time, the clinical course is silent and slowly progressive, but in rare cases it could be characterized by lymph nodes and internal organs’ involvement, with prognosis and quality of life decay [2,3]. According to Brambilla et al. staging, KS can be classified in non-advanced and advanced clinical forms: non-advanced clinical forms are characterized by exclusively cutaneous and subcutaneous involvement; furthermore, these clinical forms can be divided into locally non-aggressive and locally aggressive. Instead, advanced or disseminate clinical forms are characterized by visceral involvement [2]. Numerous treatments are used in therapeutic strategies for non-advanced KS lesions: surgical excision (excisional biopsy), cryotherapy, radiotherapy, intralesional chemotherapy, and the use of isotretinoin gel [4,5]. Systemic and locoregional therapies or radiotherapy represent the main options for the treatment of the disease, but, however, they are not always feasible due to the development of tumor resistance or the deterioration of the patient’s performance status (PS). Therefore, the alternatives we can rely on include photodynamic, intralesional, and topical therapies, which have shown a high degree of effectiveness, although they still lack standardized protocols to optimize their application [6,7]. Unfortunately, none of these could be considered effective in terms of curative response [8]. Over the last two decades, there has been a notable breakthrough in the development of new treatment modalities, including one which combines transient tumor permeabilization achieved by appropriately tuned electrical impulses with cytotoxic agents (electrochemotherapy, ECT) [7,8,9,10]. Nowadays, electrochemotherapy (ECT) has reached the greatest interest for the treatment of cutaneous and subcutaneous lesions in non-advanced KS patients (stage I and slowly progressive stage II) [11]. Electrochemotherapy (ECT) is a non-thermal tumor ablation technique which uses high-intensity pulsed electric fields to temporarily increase cell membrane permeability through the creation of pores, by which small molecules can diffuse inside cells before closing again [12,13,14,15,16]; it combines the phenomenon of electroporation with the administration of highly cytotoxic chemotherapy [8,12,13,14,15,16]. The electrical impulses induced through specific electrodes lead to the transient opening of pores on the cell membrane, and all this favors the massive entry of drug molecules into the cytosol. Therefore, the main advantage of ECT is precisely the intensity of the local dose, obtained through a high intra-tumoral concentration of the drug [7,14]. The drugs most used in ECT are Bleomycin and Cisplatin [11,12,13,14,15,16]; in fact, their cytotoxicity appears to be increased by 1000 and 80 times, respectively, in the presence of the electroporation phenomenon. In addition to drug-induced cellular destruction, electroporation appears to be responsible for vascular changes within the tumor: a “vascular lock” is induced, with a temporary reduction in perfusion in the tumor tissue with an interstitial edema. The aim of our study is not only to validate and confirm that ECT represents the best therapeutical choice in terms of the risk–benefit ratio for the treatment of cutaneous and subcutaneous lesions in non-advanced forms of KS but also to demonstrate the valid use of ECT for the local control of locally advanced CKS.

## 2. Materials and Methods

This was a retrospective single-center study enrolling 20 patients with classic KS lesions (15 males and 5 females) with median age at diagnosis of 72 (Table 1). They were referred to the Division of Plastic Surgery at the I.R.C.C.S.-Centro di Riferimento Oncologico della Basilicata, Rionero in Vulture, Italy, from November 2018 to December 2022.

The final goal of this study was the assessment of the clinical effectiveness rate; the other purposes were the evaluation of side effects, local control of the neoplasm, and the quality of life impact. Patients’ enrollment was carried out according to the Brambilla CKS staging system [17,18] and according to the ESOPE criteria [19]. To be included in this study, patients must satisfy some criteria, as follows: KS histological diagnosis with cutaneous and subcutaneous lesions which cannot be healed by local treatments like surgery, radiotherapy, or intralesional vincristine; no extracutaneous involvement demonstrated by diagnostic procedures; age > 18 years; Karnowski performance status > 70; and a washout period of at least 4 weeks after previous treatments [8]. Selected patients were not eligible for all other standard therapeutic options or had already undergone them without obtaining any benefit for their clinical condition (surgery, radiotherapy, isolated limb infusion/perfusion, chemotherapy, immunotherapy). Among the exclusion criteria we have patients who have previously shown allergic and anaphylactoid reactions to bleomycin or to any component necessary for sedation, patients who have exceeded the maximum cumulative dose of 250 mg of bleomycin/m^2^ (400,000 IU bleomycin/m^2^), and patients who had chronic renal dysfunction (serum creatinine > 150 mmol/L) or acute lung infection. Therefore, patients could be excluded if they presented abnormal respiratory parameters, cardiac pacemaker or arrhythmias, or history of seizures [7]. Only 1 male patient was excluded from the study because of comorbidities. A total of 14 patients were classified as stage I, while the other 5 patients were classified as stage II (Table 1). Written informed consent was approved by every patient so that they could be a part of the study; numerous patients’ epidemiologic distinguishing marks have been collected (ethnic group, age at arising lesions, gender), as well as clinical markers (location of the lesions, treatments carried out, clinical response, and time at disease relapse during follow-up). All of them underwent diagnostic confirmation through punch biopsy. Because of multiple or disseminated cutaneous and subcutaneous lesions mainly located in the lower limbs, many patients should not undergo excisional biopsy, radiotherapy, or topic chemotherapy; instead, these patients underwent ECT treatment. Multiple lesions’ patients who were too difficult to treat in a single session or patients who obtained a partial response (PR) at a first ECT application underwent repeated treatments. Treatments were made at the IRCCS CROB with the CliniporatorTM device (IGEA, Modena, Italy). The anesthesiology technique was chosen through the ESOPE criteria, but single patient’s adaptations were allowed according to local protocols. Each patient underwent general anesthesia or spinal anesthesia. Various electrodes were used: a type I electrode, made up of two parallel stainless-steel plates with varying distances between 6 and 8 mm, was employed for small superficial lesions’ management; a type II electrode, made up of two parallel arrays of needles with a 4 mm gap, was employed for small nodules’ management; and a type III electrode, made up of a hexagonal array of needles with an 8 mm gap, was employed for big nodules’ management. According to the AIFA note regarding drugs with consolidated use in the treatment of solid tumors in adults for indications even different from those provided by the marketing authorization measure [20], patients were previously treated with intravenous Bleomycin, promoting a homogeneous drug concentration. Intravenous Bleomycin was injected at a dose of 15,000 UI/m^2^; electric pulses were carried out 8 min after Bleomycin infusion, obtaining the most favorable drug concentration in tissues. Treated lesions were studied on day 0 and followed-up at weeks 4, 8, 12, and every 3 to 6 months thereafter. Lesions’ sizes were established by measuring the largest diameter of the lesion; clinical response and the treatment’s efficacy were assessed according to the RECIST criteria [21], as follows: progressive disease (PD), if lesions increase in the larger diameter of >20%; partial response (PR), if lesions decrease by 30% after a 4-week follow-up; unchanged clinical situation, if lesions increase by <20% or decrease by <50%; and complete response (CR), if all lesions have completely disappeared. Treatments’ systemic toxicity and side effects were evaluated according to the World Health Organization’s criteria. The impact of lifestyle was assessed through Patient Global Assessment (PGA) [22]. Statistical evaluation was performed by Minitab Analytics software 21.3 (© 2022 Minitab, LLC. All Rights Reserved, State College, PA, USA). Contingency tables and the chi-square test were used as tests for the evaluation of the different response of the lesions after ECT treatments. Kaplan–Meier analysis was used as a method to assess the local control of the disease over time. It was evaluated measuring the period from the successful ECT treatment response to either relapse’s appearance in CR results or >25% size augmentation in PR results, or last follow-up medical examination. Time to treatment failure was studied from the first day of treatment with ECT to either tumor relapse necessitating another available therapy, treatment interruption, or death from any cause. Overall survival was assessed from the first day of treatment with ECT to either death from any cause or last date of follow-up, counting all deaths as events.

## 3. Results

### 3.1. ECT Treatments

**Figure 1:**
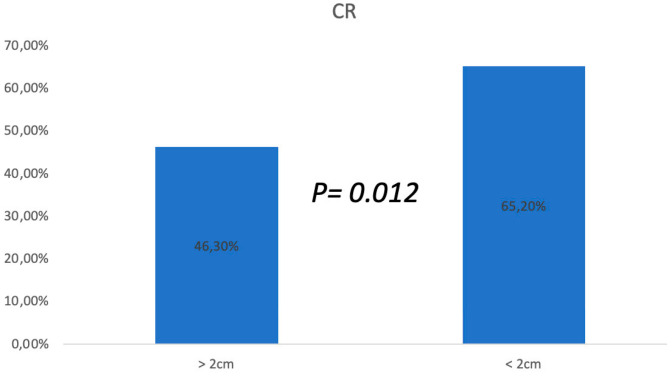
ECT success rate in KS cutaneous and subcutaneous different lesions.

In this study, a total of 19 patients with widespread and persistent KS cutaneous lesions were enrolled and underwent ECT treatments. According to the RECIST criteria [21], a clinical response, evaluated after 4 weeks, was gained by all patients, but a complete regression (CR) was obtained in 12 of 19 cases (63.2%), and a partial regression (PR) was obtained in the other 7 cases (36.8%) (Table 1). No differences have been demonstrated among the overall response rate according to the size of the cutaneous lesions, but the CR rate of bigger lesions (>2 cm) was lower than the CR rate of smaller lesions (46.3% vs. 65.2%; χ^2^ test, *p* = 0.012) (Figure 1). 

After the first ECT session, seven patients (36.8%) received a second ECT treatment; three of seven patients gained CR, but the other four cases presented PR (Table 1). Furthermore, these four cases underwent a third ECT treatment and subsequently achieved PR with respect to the initial measurement. Therefore, ECT treatments led to a complete regression in 15 of 19 patients (78.9%), in 12 patients after the first ECT attempt and in 3 patients after the second attempt (Table 1). The mean interval between the two treatments was 90 days.

### 3.2. Follow-Up

**Figure 2:**
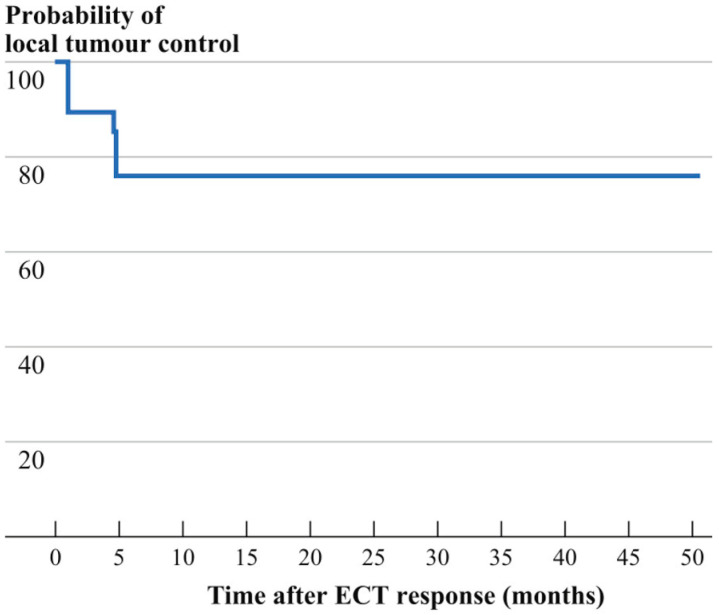
Local control of the disease in 19 CKS patients who underwent ECT.

Clinical response was observed during the entire time of the follow-up, which ranged between 3 months and 4 years, with a median of 18 months. CR was obtained in 10 cases with stage I, in 5 cases with stage II, and in 0 stage III or IV cases (Table 1). After a median follow-up of 18 months, 15 patients maintained their response, but only 3 cases achieved this after repeated courses. Meanwhile, the other four PR patients either were not retreated with ECT or they underwent further therapies because of the silent course of the KS classic variant and the excellent impact of the disease on quality of life. (Figure 2).

### 3.3. Side Effects and Quality of Life

Among the side effects and quality of life, the treatment was well tolerated, with a very low complication rate. Pain and erythema were among the most reported side effects; cutaneous infections were noticed in only three patients, and they were healed with oral antibiotics, causing complete healing in less than a few days. No other symptoms nor systemic toxicity occurred. According to PGA [22], in terms of quality of life, an improvement was obtained in all patients; this was related to an excellent result after treatment.

## 4. Discussion

The widespread presence of cutaneous and subcutaneous CKS lesions is an uncomfortable situation for many patients. The presence of multiple skin and subcutaneous lesions worsens patients’ quality of life, frequently burdened by pain, ulceration, and bleeding. Their management and treatment are an arduous challenge for operators as they often depend on the number and size of the lesions, their anatomical sites, and the appearance of visceral lesions. When possible, definitive surgical removal is the most suitable therapeutic approach. However, when surgery cannot be performed due to an excessive number of lesions and their considerable extension with the compromise of a reasonable result from a functional point of view, other therapeutic options must be considered [1,7,14,23,24]. Unfortunately, there is no standard therapy policy for CKS [25]. According to the Brambilla et al. staging system [18], in which each patient can be classified into four stages, namely I—macular-nodular, II—infiltrative, III—florid, and IV—disseminate, respectively, and further divided into two categories (A and B, based on the evolution rate of the disease), an accurate CKS patient’s staging allows for the choice of the most appropriate therapeutic strategy [2,24]. After clinical staging, the patient should undergo specialist visits and instrumental investigations to exclude evidence of internal organs’ involvement [2,26].

Although complications are mainly frequent in advanced stages (stage III and IV), functional impairment and pain or lymphoedema, bleeding, and ulceration can occur in all stages of the disease [2,18]. Visceral involvement, if present, usually occurs in the context of stage III or IV disease. Therefore, only after the correct staging of the patient can the diagnostic–therapeutic process can continue. Most CKS cutaneous and subcutaneous lesions treated are symptomatic lesions, unlike asymptomatic or slowly evolving ones, for which a wait-and-see strategy can be chosen, because a spontaneous improvement could occur [4,5]. Therapeutic strategies are chosen after the assessment of the tumor staging, the localization and the evolution pattern of the lesions, clinical type, and immune status [6,7,8,9,10]. Local treatments should be performed alone in localized KS, or in combination with systemic treatments in patients with advanced or disseminated KS [2,4].

**Figure 3:**
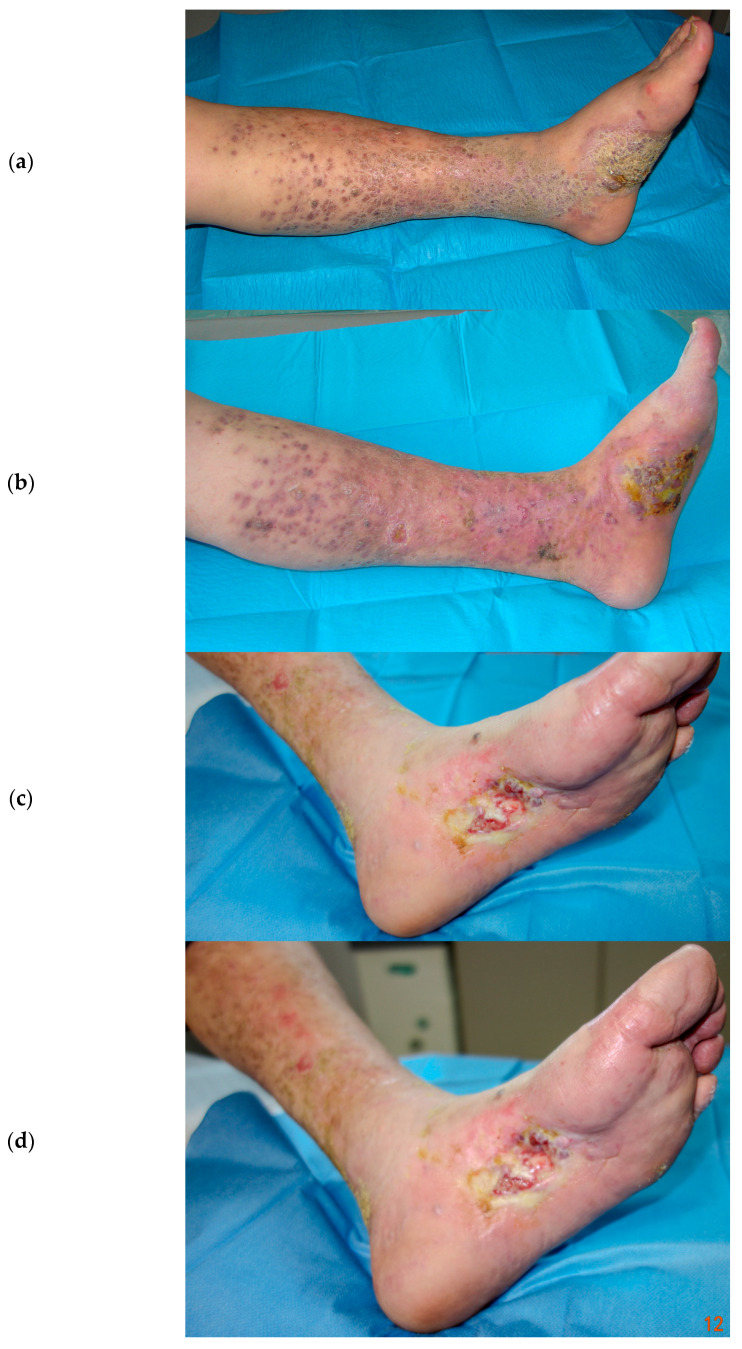
(**a**) Left leg widespread skin and subcutaneous Kaposi lesions in a 63-year-old patient. Before ECT treatment. (**b**) After three ECT treatments with systemic Bleomycin. (**c**,**d**) Local control of the disease after 3 years and 4 years follow-up, respectively.

**Figure 4:**
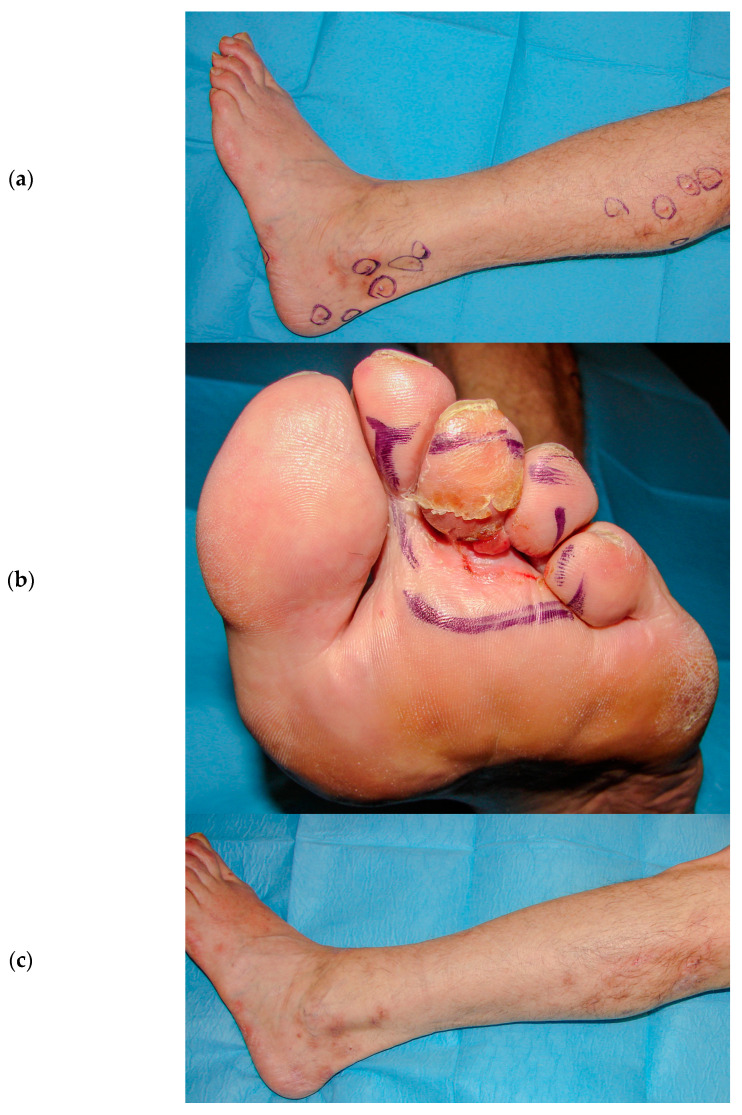

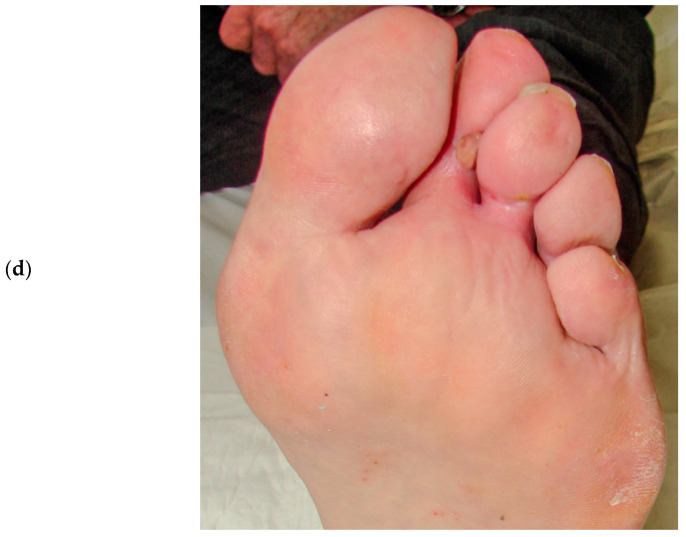
(**a**,**b**) Widespread plaques and nodules on the leg and foot. (**c**) Complete remission of healed nodules and plaques with residual hyperpigmentation and hypotrophic scars. (**d**) Partial remission of treated lesion with residual nodule.

Surgical excision should be performed exclusively on a couple and well-defined lesions; also, radiotherapy or intralesional injections are included in local treatments. Despite the poor toxicity pattern induced by these therapies, their application on several lesions is not possible because they can shatter the global status of the patients, who necessitate the preservation of their immunocompetent status, meaning that many of these patients would have received systemic treatments, including single or combined antitumoral drugs (vinca alkaloids, etoposide, liposomal doxorubicin, bleomycin) [2]. This study shows the results of a retrospective trial aimed to evaluate the clinical response and toxicities of ECT with an intravenous bleomycin injection among the treatments of CKS cutaneous and subcutaneous lesions, demonstrating that ECT is non-inferior to other local and systemic treatments. In fact, clinical response was gained in all cases, apart from tumor dimensions, with 63% of cases resulting in a CR after the first ECT attempt; moreover, other additional CRs were gained after repeated sessions. Despite the good overall response rates, greater CR rates were obtained by patients with nodules or plaques with <2 cm diameter (65.2% vs. 46.3%). In this study, ECT, after bleomycin was previously administered, obtained positive results in the local control of the disease, whereas other treatments, such as surgery or radiotherapy, would have been hazardous because of the high risk of side effects like ulceration, bleeding, infection, and delayed healing. Because ECT has already been discussed by several authors as a therapy growing in popularity for the treatment of cutaneous neoplasms also in first-line choices, it is worth extending the indications of ECT therapy to clinical situations that cannot otherwise be treated, such as other skin tumors, for example, unresectable metastases in melanoma or breast cancer or cylindromas of the scalp, as a adjuvant or palliative treatment [27,28,29]. Furthermore, we observed in our experience that ECT could be successful also in the management of the disease for those patients affected by advanced and widespread CKS cutaneous lesions (Figure 3 and Figure 4).

Almost all patients presented pain and erythema after treatment, and only three cases reported infections, which were then treated with antibiotics. No systemic toxicities occurred, compared to the higher toxicity which occurred due to classical treatments. Furthermore, an improvement in quality of life is noticed, as established by the PGA [22], not only related to the stage of the disease.

## 5. Conclusions

Based on several studies previously published in the literature [7,9,14,27,28,29,30,31,32], this single-center study strongly supports the efficacy of ECT in patients with superficial cutaneous and subcutaneous KS lesions, and in the same way, it could also be used for the control of locally advanced lesions. ECT generated a strong and long-lasting clinical response, with the development of very limited toxicity; in fact, it seems to preserve and even dramatically improve health-related quality of life. Therefore, all this has translated into a very high acceptance rate among patients. Therefore, all ECT candidates can also undergo treatment with locoregional anesthesia, thus confirming the easy management of this treatment and, above all, that it is a treatment well tolerated by patients. Because of our and others’ experiences, we can affirm that ECT could be considered a valid therapeutical strategy for the local control of widespread and advanced CKS cutaneous and subcutaneous lesions, and that it is the gold-standard therapy in non-advanced CKS patients. In conclusion, the clinical application of ECT is growing, and the results are promising.

## Figures and Tables

**Table 1:** Patients’ characteristics and results after 18 months. CR: complete response; PR: partial response.

Patients n°	Sex, Age	Localization	Clinical Response	Response	Stage
1	F, 85	Right foot	Present	CR	I
2	F, 74	Lower limb	Present	CR at second ECT	I
3	F, 63	Lower limbs bilateral Foot	Present	PR after third ECT	II
4	F, 55	Foot	Present	CR	I
5	F, 70	Lower limbs bilateral Foot	Present	CR	I
6	M, 72	Foot	Present	CR at second ECT	II
7	M, 68	Foot	Present	CR	I
8	M, 60	Foot	Present	CR	I
9	M, 80	Lower limbs	Present	CR	I
10	M, 75	Right foot	Present	CR	I
11	M, 71	Bilateral foot	Present	PR after third ECT	II
12	M, 78	Lower limbs	Present	CR	I
13	M, 77	Lower limbs	Present	CR at second ECT	I
14	M, 68	Foot	Present	CR	I
15	M, 70	Lower limbs	Present	PR after third ECT	II
16	M, 76	Left limb	Present	CR	I
17	M,74	Right limb	Present	CR	I
18	M, 84	Foot	Present	CR at second ECT	I
19	M, 69	Bilateral foot	Present	PR after third ECT	II
20	M, 71	Excluded for comorbidities			

## Data Availability

The datasets used and/or analyzed during the current study are available from the corresponding author on reasonable request.

## References

[1] Etemad S.A., Dewan A.K. (2019). Kaposi Sarcoma Updates. Dermatol. Clin..

[2] Brambilla L., Genovese G., Berti E., Peris K., Rongioletti F., Micali G., Ayala F., DELLA Bella S., Mancuso R., Pinton P.C. (2021). Diagnosis and treatment of classic and iatrogenic Kaposi’s sarcoma: Italian recommendations. Ital. J. Dermatol. Venerol..

[3] Cox F.H., Helwig E.B. (1959). Kaposi’s sarcoma. Cancer.

[4] Dupin N. (2020). Update on oncogenesis and therapy for Kaposi sarcoma. Curr. Opin. Oncol..

[5] Chak L.Y., Gill P.S., Levine A.M., Meyer P.R., Anselmo J.A., Petrovich Z. (1998). Radiation therapy for acquired immunodeficiency syndrome-related Kaposi’s sarcoma. J. Clin. Oncol..

[6] Toschi E., Sgadari C., Monini P., Barillari G., Bacigalupo I., Palladino C., Baccarini S., Carlei D., Grosso G., Sirianni M.C. (2002). Treatment of Kaposi sar- coma-an update. Anticancer. Drugs..

[7] Campana L., Testori A., Curatolo P., Quaglino P., Mocellin S., Framarini M., Borgognoni L., Ascierto P., Mozzillo N., Guida M. (2016). Treatment efficacy with electrochemotherapy: A multi-institutional prospective observational study on 376 patients with superficial tumors. Eur. J. Surg. Oncol..

[8] Curatolo P., Quaglino P., Marenco F., Mancini M., Nardò T., Mortera C., Rotunno R., Calvieri S., Bernengo M.G. (2012). Electrochemotherapy in the treatment of Kaposi sarcoma cutaneous lesions: A two-center prospective phase II trial. Ann. Surg. Oncol..

[9] Testori A., Tosti G., Martinoli C., Spadola G., Cataldo F., Verrecchia F., Baldini F., Mosconi M., Soteldo J., Tedeschi I. (2010). Electrochemotherapy for cutaneous and subcutaneous tumor lesions: A novel therapeutic approach. Dermatol. Ther..

[10] Garbay J.R., Billard V., Bernat C., Mir L.M., Morsli N., Robert C. (2006). Successful repetitive treatments by electrochemotherapy of multiple unresectable Kaposi sarcoma nodules. Eur. J. Cancer.

[11] Latini A., Bonadies A., Trento E., Bultrini S., Cota C., Solivetti F.M., Ferraro C., Ardigò M., Amorosi B., Palamara G. (2012). Effective treatment of Kaposi’s sarcoma by electrochemotherapy and intravenous bleomycin administration. Dermatol. Ther..

[12] Giardino R., Fini M., Bonazzi V., Cadossi R., Nicolini A., Carpi A. (2006). Electrochemotherapy a novel approach to the treatment of metastatic nodules on the skin and subcutaneous tissues. Biomed. Pharmacother..

[13] Mir L.M., Gehl J., Sersa G., Collins C.G., Garbay J.-R., Billard V., Geertsen P.F., Rudolf Z., O’sullivan G.C., Marty M. (2006). Standard operating procedures of the electrochemotherapy: Instructions for the use of bleomycin or cisplatin administered either systemically or locally and electric pulses delivered by the Cliniporator^TM^ by means of invasive or non-invasive electrodes. Eur. J. Cancer Suppl..

[14] Solari N., Spagnolo F., Ponte E., Quaglia A., Lillini R., Battista M., Queirolo P., Cafiero F. (2014). Electrochemotherapy for the management of cutaneous and subcutaneous metastasis: A series of 39 patients treated with palliative intent. J. Surg. Oncol..

[15] Miklavcic D., Snoj M., Zupanic A., Kos B., Cemazar M., Kropivnik M., Bracko M., Pecnik T., Gadzijev E., Sersa G. (2010). Towards treatment planning and treatment of deep-seated solid tumors by electrochemotherapy. Biomed. Eng. Online.

[16] Gehl J. (2003). Electroporation: Theory and methods, perspectives for drug delivery, gene therapy and research. Acta Physiol. Scand..

[17] Chachoua A., Krigel R., Lafleur F., Ostreicher R., Speer M., Laubenstein L., Wernz J., Rubenstein P., Zang E., Friedman-Kien A. (1989). Prognostic factors and staging classification of patients with epidemic Kaposi’s sarcoma. J. Clin. Oncol..

[18] Brambilla L., Boneschi V., Taglioni M., Ferrucci S. (2003). Staging of classic Kaposi’s sarcoma: A useful tool for therapeutic choices. Eur. J. Dermatol..

[19] Marty M., Sersa G., Garbay J.R., Gehl J., Collins C.G., Snoj M., Billard V., Geertsen P.F., Larkin J.O., Miklavcic D. (2006). Electrochemotherapy an easy, highly effective and safe treatment of cutaneous and subcutaneous metastases: Results of ESOPE (European Standard Operating Procedures of Electrochemotherapy) study. Eur. J. Cancer Suppl..

[20] DeVita V.T., Lawrence T.S., Rosenberg S.A. (2000). Cancer: Principles and Practice of Oncology.

[21] Therasse P., Arbuck S.G., Eisenhauer E.A., Wanders J., Kaplan R.S., Rubinstein L., Verweij J., Van Glabbeke M., van Oosterom A.T., Christian M.C. (2000). New guidelines to evaluate the response to treatment in solid tumors. European Organization for Research and Treatment of Cancer, National Cancer Institute of the United States, National Cancer Institute of Canada. J. Natl. Cancer Inst..

[22] Pincus T., Bergman M., Sokka T., Roth J., Swearingen C., Yazici Y. (2008). Visual analog scales in formats other than a 10 centimeter horizontal line to assess pain and other clinical data. J. Rheumatol..

[23] Sullivan R.J., Pantanowitz L. (2010). New drug targets in Kaposi sarcoma. Expert. Opin. Ther. Targets..

[24] Brambilla L., Miedico A., Ferrucci S., Romanelli A., Brambati M., Vinci M., Tedeschi L., Boneschi V. (2006). Combination of vinblastine and bleomycin as first line therapy in advanced classic Kaposi’s sarcoma. J. Eur. Acad. Dermatol. Venereol..

[25] Radu O., Pantanowitz L. (2013). Kaposi sarcoma. Arch. Pathol. Lab. Med..

[26] Krigel R.L., Laubenstein L.J., Muggia F.M. (1983). Kaposi’s sarcoma: A new staging classification. Cancer Treat. Rep..

[27] Fabrizio T., Cagiano L., De Terlizzi F., Grieco M.P. (2020). Neoadjuvant treatment by ECT in cutaneous malignant neoplastic lesions. J. Plast. Reconstr. Aesthet. Surg..

[28] Łapińska Z., Saczko J. (2022). Novel electroporation-based treatments for breast cancer. Adv. Clin. Exp. Med..

[29] Bonadies A., Bertozzi E., Cristiani R., Govoni F.A., Migliano E. (2019). Electrochemotherapy in Skin Malignancies of Head and Neck Cancer Patients: Clinical Efficacy and Aesthetic Benefits. Acta Derm. Venereol..

[30] Mali B., Jarm T., Snoj M., Sersa G., Miklavcic D. (2013). Antitumor effectiveness of electrochemotherapy: A systematic review and meta-analysis. Eur. J. Surg. Oncol..

[31] Di Monta G., Caracò C., Benedetto L., La Padula S., Marone U., Tornesello M., Buonaguro F., Simeone E., Ascierto P., Mozzillo N. (2014). Electrochemotherapy as “new standard of care” treatment for cutaneous Kaposi’s sarcoma. Eur. J. Surg. Oncol..

[32] Heller R., Jaroszeski M.J., Reintgen D.S., Puleo C.A., DeConti R.C., Gilbert R.A., Glass L.F. (1998). Treatment of cutaneous and subcutaneous tumors with electrochemotherapy using intralesional bleomycin. Cancer.

